# A prognostic scoring system for operated acute epidural hematoma based on gray–white matter ratio

**DOI:** 10.1097/MD.0000000000026888

**Published:** 2021-08-20

**Authors:** Yunxing Luo, Xiwu He, Mingfei Yang, Chaonan Du, Xiaoqing Jin

**Affiliations:** a Graduate School, Qinghai University, Xining, Qinghai 810016, PR China; b Department of Neurosurgery, the Fifth People's Hospital of Qinghai Province, Xining, Qinghai 810007, PR China; c Department of Neurosurgery, Qinghai Provincial People's Hospital, Xining, Qinghai 810007, PR China.

**Keywords:** acute epidural hematoma, computed tomography, gray–white matter, nomogram, prognosis

## Abstract

To determine the prognostic risk factors of patients with acute epidural hematoma (AEDH), a scoring system was established based on gray–white matter ratio (GWR) and internal verification was performed.

All patients with AEDH who underwent surgical treatment in Qinghai Provincial People's Hospital from January 2013 to June 2019 were continuously collected. The clinical and imaging data of the patients were collected. According to Glasgow Outcome Scale at 3 months after operation, the patients were divided into poor and good prognosis groups, respectively. The GWR value of the nonhematoma side was measured at the inner capsule area. Univariate and multivariate analyses were used. Independent predictors significantly related to the prognosis of AEDH were screened out and a nomogram was established based on these factors.

A total of 170 cases were included in this study, the Glasgow Coma Score (severe and moderate), cerebral hernia, midline shift, preoperative GWR, postoperative GWR, hematoma thickness/midline shift, time from coma to surgery, and decompression of bone flap were the independent risk factors for predicting the poor prognosis of AEDH. Moreover, the prediction ability of nomogram was higher than any other independent predictive factors.

The nomogram model established represents the most effective factor to predict the prognosis of operated AEDH. The scoring system is characterized by high accuracy, simplicity and feasibility, with a wide range of clinical application prospects.

## 1 Introduction

Traumatic brain injury (TBI) is one of the main causes of accidental death among the under-45-year-old population.^[1]^ Acute epidural hematoma (AEDH) is one of the most common types of TBI, accounting for 2.7% to 11.0% of all TBI patients.^[2]^ The patients with large hematoma are prone to suffer from cerebral hernia in the early stage, and an emergency surgical intervention would be necessary. Most of the patients with AEDH recover well, but 10% would still die.^[3]^ Therefore, it is particularly important to evaluate the prognosis of AEDH patients at an early stage.

Rotterdam computed tomography (CT) score^[4]^ and Helsinki CT score^[5]^ have been often used to evaluate the prognosis of TBI in clinical practice. However, these scoring systems have some shortcomings. For example, the Rotterdam CT scoring system only describes the midline shift (MLS) and compression state of basal cistern, which would fail to take into account the specific characteristics of a single disease case. Guo et al^[6]^ have proposed the effect of swirl sign on prognosis in AEDH, while no other features of AEDH have been investigated. At present, there is only 1 scoring system for acute subdural hematoma,^[7]^ while there is no scoring system for AEDH. Therefore, in this study, based on previous findings, a scoring system for the prognosis of AEDH was proposed, and the internal verification was carried out.

## 2 Materials and methods

### 2.1 Ethical review

This study was approved by the ethics committee of Qinghai Provincial People's Hospital. Informed consent was obtained from each patient.

### 2.2 Patient selection

All patients with AEDH who underwent surgical treatment in Qinghai Provincial People's Hospital from January 2013 to June 2019 were continuously collected.

### 2.3 Inclusion criteria

Subjects more than 18 years old; diagnosed with unilateral AEDH by CT, located on the superior side of the cerebellar tentorium, representing the primary life-threatening cause; with abbreviated injury scale (AIS) (other than the head) less than 3 points; epidural hematoma was cleared, and decompression of bone flap was determined according to the situation during operation; and with no history of craniocerebral surgery or epilepsy.

### 2.4 Exclusion criteria

Patients with cerebral infarction, cerebral hemorrhage or intracranial occupying lesion in the past; with cancer or other serious diseases; with incomplete clinical, image or follow-up data; with penetrating brain injuries; and with any disease that would affect the coagulation function, such as coagulopathy, renal dysfunction, anticoagulant therapy, antiplatelet therapy, etc.

### 2.5 Data collection

The general data mainly included the sex, age, nationality, occupation, past medical history, treatment, cerebral hernia, AIS, Glasgow Coma Score (GCS), time from coma to surgery, and Glasgow Outcome Scale (GOS). The imaging data mainly included the hematoma volume, state of basal cistern, hematoma thickness (HT)/MLS, and gray matter (GM) and white matter (WM) Hounsfield values in nonhematoma side. Gray–white matter ratio (GWR) was obtained according to the preoperative and the first postoperative CT images, according to the following formulation: GWR = GM/WM.

### 2.6 Subject grouping

The patients were divided into a poor prognosis group (GOS 1–2 points) and a good prognosis group (GOS 3–5 points), respectively, according to the postoperative 3-month GOS.

### 2.7 CT assessment

CT scanning was performed using a unified protocol, with the equipment from the General Electric Company (USA) (the axial scanning thickness was 5 mm). Based on the medical imaging information management system (version 2.0), the circular region of interest was placed independently in the measurement area by a radiologist and a neurosurgeon. The original CT values of GM and WM were measured in the region of interest.

### 2.8 Calculation of GWR

**Figure 1:**
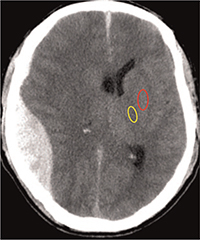
Gray matter measure area (red circle) and white matter measure area (yellow circle).

GWR of the nonhematoma side was measured at the inner capsule area. According to the GWR calculation method reported by Gentsch et al,^[8]^ the putamen (PU) was selected as the GM measurement area and the posterior limb of internal capsule (PIC) was selected as the WM measurement area. However, the PU and pallidus (PA) were not clear, and therefore, the lentiform nucleus (LN) was used as the measurement area in this study (Fig. 1). The calculation formulation was modified into the following: GWR = LN/PIC.

### 2.9 Treatment plan

All patients were treated with the standard operative approach rather than the minimally invasive one,^[9,10]^ and the decompression of bone flap was determined according to the situation during operation. Postoperative treatment was conducted according to the TBI guideline.^[11]^

### 2.10 Statistical analysis

The receiver operating characteristic (ROC) curve was plotted using the graphics printer. According to the ROC curve, the continuous variables were evaluated, and the cutoff value was determined. The SPSS software (version 24.0; IBM, Armonk, NY) was used for the univariate and multivariate logistic regression analyses. All factors associated with poor prognosis were assessed using the *P* values, odds ratios (OR), and 95% confidence intervals (CI). The Chi-square test was used for univariate analysis to preliminarily identify risk factors associated with poor prognosis. The significant risk factors in the univariate analysis were analyzed by the binary logistic regression analysis. *P* < .05 was considered statistically significant.

Using the R software version 3.4.3, the forest map and nomogram were drawn based on the results from the binary logistic regression analysis. The predictor with the highest beta coefficient was assigned 100 points based on the weight, and the other predictors were assigned corresponding points. In order to prove the net benefit of the established nomogram prediction score system, the decision curve was used to analyze the independent risk factors and the nomogram prediction scoring system. Clinical influence curve was used to verify the accuracy of the above decision curve analysis.

## 3 Results

### 3.1 ROC curve analysis

A total of 632 patients were enrolled in this study. Among these subjects, 170 cases met the inclusion criteria, while 462 cases were excluded (17 patients were less than 18 years old, 248 patients had incomplete clinical and imaging data, 35 patients had infratentorial hematomas, 72 patients had bilateral hematomas, and 90 patients were with AIS ≥ 3 points).

**Figure 2:**
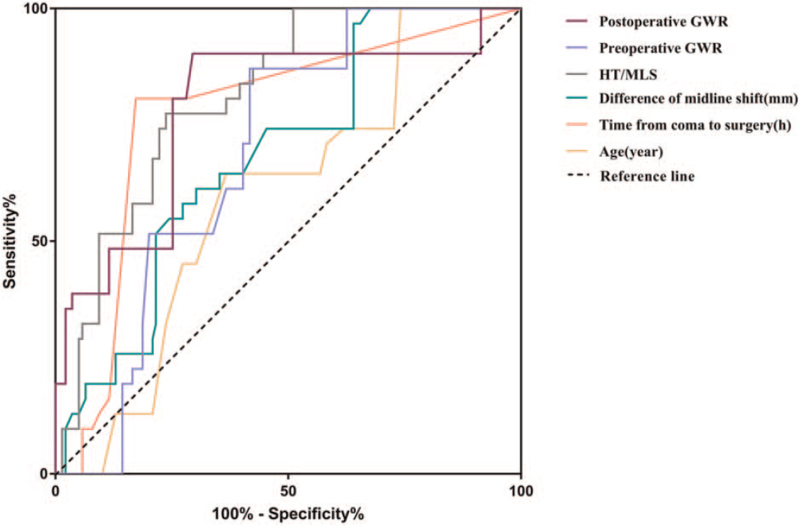
Receiver operating characteristic curve analysis. GWR = gray–white matter ratio, HT = hematoma thickness, and MLS = midline shift.

In the ROC analysis, the factors of age, difference of MLS (the difference between the preoperative and postoperative MLS), HT/MLS, preoperative GWR, postoperative GWR, and the time from coma to surgery were analyzed. Our results showed that the area under curve values of all the above factors was more than 50%, and the area under curve value of HT/MLS was the largest at 81.0% (Fig. 2).

### 3.2 Analysis of influencing factors of poor prognosis

#### 3.2.1 Univariate analysis

Based on the univariate analysis, at the significant level of *P* = .05, the factors of age, state of basal cistern, GCS, cerebral hernia, difference of MLS, preoperative GWR, postoperative GWR, HT/MLS, time from coma to surgery, and decompression of bone flap were correlated with poor prognosis (Table 1). Accordingly, the multivariate analysis of these factors was then carried out.

**Table 1:** Univariate analysis.

		Prognosis				
Variables	Subunit	Good	Poor	Rate of poor prognosis (%)	χ^2^	*P*
Sex	Man	132	27	16.98	2.592	.107
	Woman	7	4	36.36		
Age (year)	≤43	92	10	9.80	12.158	<.001
	>43	47	21	30.88		
Occupation	Peasant	82	24	22.64	3.991	.136
	Worker	10	2	16.67		
	Other	47	5	9.62		
Nationality	Han	85	23	21.30	3.592	.166
	Tibetan	25	6	19.35		
	Other	29	2	6.45		
State of basal cistern	Normal	102	14	12.07	9.313	.002
	Compressed	37	17	31.48		
Decompression of bone flap	Yes	37	29	43.94	47.806	<.001
	No	102	2	1.92		
Cerebral hernia	Yes	35	29	45.31	50.472	<.001
	No	104	2	1.89		
Tracheotomy	Yes	33	11	25.00	1.822	.177
	No	106	20	15.87		
Time from coma to surgery (h)	≤4.5	121	11	8.33	38.833	<.001
	>4.5	18	20	52.63		
GCS	Severe	19	17	47.22	39.316	<.001
	Moderate	20	10	33.33		
	Mild	100	4	3.85		
Difference of midline shift (mm)	≤2.9	69	3	4.17	16.580	<.001
	>2.9	70	28	28.57		
HT/MLS	≤2.52	45	3	6.25	6.444	.011
	>2.52	94	28	22.95		
Postoperative GWR	≤1.368	41	28	40.58	38.890	<.001
	>1.368	98	3	2.97		
Preoperative GWR	≤1.307	58	27	31.76	20.870	<.001
	>1.307	81	4	4.71		

GCS = Glasgow Coma Score; GWR = gray–white matter ratio; HT = hematoma thickness; MLS = midline shift.

#### 3.2.2 Multivariate analysis

Our results from the multivariate analysis showed that the GCS (severe) (OR: 39.160; 95% CI: 3.001–61.921) was the most dangerous factor for poor prognosis, followed by the postoperative GWR ≤1.368 (OR: 36.994; 95% CI: 3.808–59.411). However, the state of basal cistern and age were not independent predictors of poor prognosis (Table 2).

**Table 2:** Multivariate analysis.

								95% CI of OR	
Variables	Subunit	B	S	Wald	v	*P*	OR	Minimum	Maximum
State of basal cistern	Compressed	1.055	0.836	1.590	1	.207	2.871	0.557	14.791
	Normal	0					1		
Decompression of bone flap	Yes	3.315	1.615	4.215	1	.040	27.528	1.162	51.986
	No	0					1		
Cerebral hernia	Yes	2.037	1.010	4.067	1	.044	7.670	1.059	15.554
	No	0					1		
Age (year)	>43	0.156	0.769	0.041	1	.840	1.169	0.259	5.278
	≤43	0					1		
HT/MLS	≤2.52	2.616	1.047	6.244	1	.012	13.688	1.758	56.570
	>2.52	0					1		
Time from coma to surgery (h)	>4.5	3.023	1.188	6.474	1	.011	20.556	2.003	41.007
	≤4.5	0					1		
Difference of midline shift (mm)	>2.9	2.255	0.929	5.899	1	.015	9.537	1.545	28.851
	≤2.9	0					1		
Preoperative GWR	≤1.307	2.990	1.329	5.061	1	.024	19.886	1.470	39.071
	>1.307	0					1		
Postoperative GWR	≤1.368	3.611	1.160	9.688	1	.002	36.994	3.808	59.411
	>1.368	0					1		
GCS	Severe	3.668	1.311	7.832	1	.005	39.160	3.001	61.921
	Moderate	2.224	1.114	3.987	1	.046	9.243	1.042	22.009
	Mild	0					1		
Constant	−13.711	3.163	18.793	1	0.000	.000			

GCS = Glasgow Coma Score; GWR = gray–white matter ratio; HT = hematoma thickness; MLS = midline shift.

### 3.3 Forest map analysis

**Figure 3:**
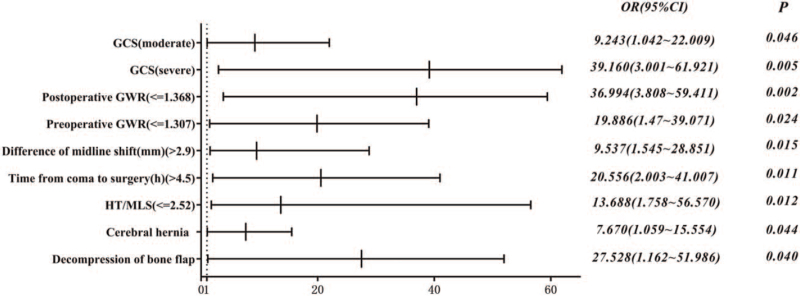
Forest map analysis. GCS = Glasgow Coma Score, GWR = gray–white matter ratio, HT = hematoma thickness, and MLS = midline shift.

Based on the results from the univariate and multivariate analyses, 8 independent predictors were identified in total which are as follows: the GCS (severe and moderate), cerebral hernia, difference of midline shift, preoperative GWR, postoperative GWR, HT/MLS, time from coma to surgery, and decompression of bone flap. The forest map analysis of the prognostic risk was then carried out (Fig. 3).

### 3.4 Nomogram analysis

**Figure 4:**
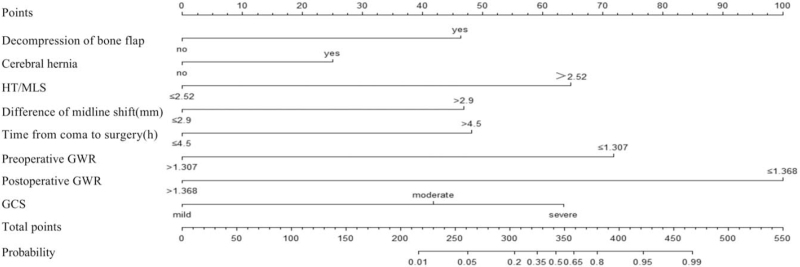
Nomogram analysis. GCS = Glasgow Coma Score, GWR = gray–white matter ratio, HT = hematoma thickness, and MLS = midline shift.

**Figure 5:**
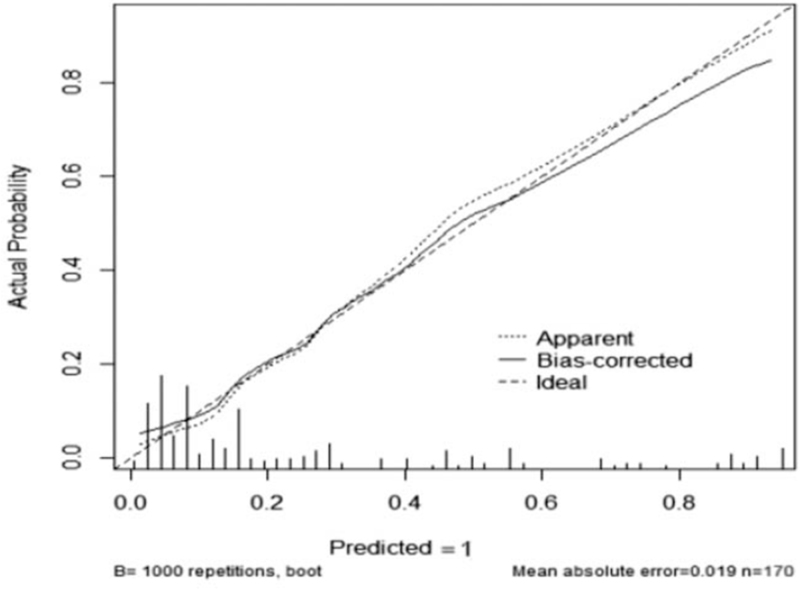
Calibration plot.

**Figure 6:**
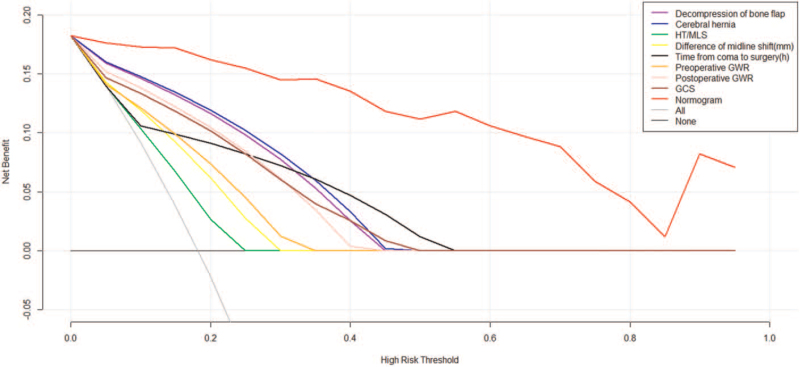
Decision curve analysis. GCS = Glasgow Coma Score, GWR = gray–white matter ratio, HT = hematoma thickness, and MLS = midline shift.

The cutoff point of 50% was adapted, that is, the patient's score was greater than 243 points, which indicated poor prognosis (Fig. 4). Our results showed that the prediction accuracy of the above scoring system was 92.24% (95% CI: 89.13%–96.57%). Furthermore, in order to verify the accuracy of the above scoring system, the calibration plot was drawn (Fig. 5). It could be seen that the prediction results and the diagonal lines were basically bonded together, suggesting very accurate prediction. Decision curve analysis proved that the net benefit of nomogram predictive scoring system was higher than any independent risk factor (Fig. 6).

**Figure 7:**
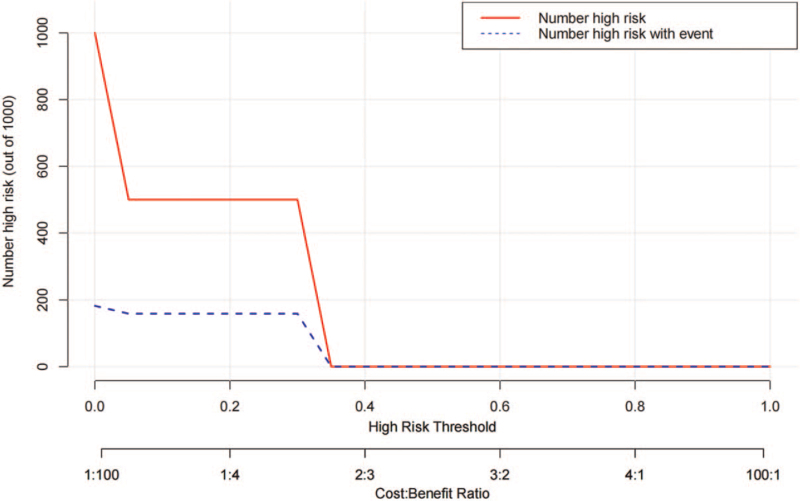
Clinical impact curve.

In the clinical influence curve, the red curve represented the number of people who were classified as positive (high risk population) according to the simple model, and the blue curve represented the number of true positive people under each threshold probability. When the threshold probability was low, the distance between the red curve and the blue curve was large, indicating significant difference in the numbers between the high-risk and actual positive people; and the opposite results indicated small difference. When the threshold probability was more than 25%, the predicted positive number was basically consistent with the actual positive number (Fig. 7).

The threshold probability of 35% was used herein, which meant that the predicted positive subjects basically matched the actual positive ones. Therefore, the nomogram scoring system was selected to evaluate the prognosis of patients with high accuracy.

### 3.5 Comparison of GM and WM between preoperative and postoperative nonhematoma side

The data were exhibiting nonnormal distribution, and therefore the median was used for statistical analysis. Our results showed that there was no difference between the preoperative and postoperative GM in the nonhematoma side, and there was significant difference between the preoperative and postoperative WM (Table 3).

**Table 3:** Comparison of GM and WM.

	Preoperative	Postoperative	Z	*P*
GM	35.53 (34.23–37.07)	36.35 (34.61–37.33)	−0.180	.857
WM	27.12 (25.75–27.99)	25.61 (24.42–27.01)	−6.661	<.001

GM = gray matter; WM = white matter.

## 4 Discussion

According to the GWR calculation method reported by Gentsch et al,^[8]^ the PU was selected as the GM measurement area and the PIC was selected as the WM measurement area. However, the PU and PA were not clear due to the hematoma compression, and therefore the LN was used as the measurement area in this study. The method might be more feasible and rigorous to calculate the GM and WM.

The haemostatic agents were used within the surgical site, which could influence the CT values.^[12]^ However, herein, the measurement areas selected were LN and PIC, which did not affect the gray–white measurement, nor influence the prognostic score.

The time from coma to surgery was one of the most important factors affecting the disease prognosis. It has been previously shown that the fatality rate of patients who undergo surgery within 4 hours after coma is 30%, while that of patients who undergo surgery after 4 hours is 90% (*P* < .0001).^[13]^ In this study, our results showed that the mortality of patients was positively correlated with time from coma to operation. In addition, our results showed that the mortality was 35% in patients with time from coma to operation more than 4 hours. The difference might be related to the differences in the sample size. It might also be correlated with the fact that Qinghai province is located on the plateau, and the people living there have relatively stronger tolerance to cerebral ischemia and hypoxia. Therefore, a control study concerning the high altitude and plain areas should be conducted to clarify the influence of hypoxia environment on prognosis in the future.

In the early stage of TBI, the cytotoxicity and angiogenic edema could co-exist, and the densities of GM and WM might be decreased.^[14]^ However, in this study, our results showed that there was no significant difference between the preoperative and postoperative CT values of GM. However, there was significant difference in the CT value between preoperative and postoperative WM, where the CT value of postoperative WM was lower than the preoperative WM. Moreover, our results showed that the WM was more easily to be affected by ischemia and hypoxia, even if the compression was relieved. It would continue to be influenced by ischemia and hypoxia, while GM was basically not impacted. This phenomenon might be related to the vascular densities of GM and WM.^[15]^ At the same time, this might be due to the significant decrease in the CT value of preoperative WM when the hematoma volume was large enough. However, the CT value of WM after removing hematoma exhibited no significant change or even be lowered, compared with before surgery.

In this study, our results showed that the prognosis was poor with the preoperative GWR ≤1.307 and/or postoperative GWR ≤1.368. Moreover, smaller ratios would indicate worse prognosis; and vice versa. These findings suggest that the prognosis of patients was worse when the GM was affected by ischemia and hypoxia than when WM was affected. The GM was the region of the nucleus, and when the nucleus was damaged, it could cause serious dysfunction, thus affecting the disease prognosis.

Since HT near the head top is larger while MLS is smaller, the HT/MLS value would be larger. Therefore, data analysis was conducted with the maximum value excluded. In this study, our results showed that the prognosis was poor with HT/MLS ≤ 2.52. Moreover, smaller ratios would indicate worse disease prognosis; and vice versa. These findings were consistent with those met in clinical practice. Because the brain tissue has certain compensation abilities, when the hematoma volume is larger, the hematoma spread around, making the HT increase not obvious. But if the amount of bleeding exceeded the compensation ability of brain tissue, it will lead to the greater MLS and the consequent smaller HT/MLS.

When difference between preoperative and postoperative MLS was found to be greater than 2.9 mm this indicated poor prognosis. This is because the preoperative hematoma volume determined the MLS size. The larger hematoma volume was, the more obvious MLS was, and after the hematoma was cleared, MLS is reduced, and the difference of MLS became larger. Therefore, the larger MLS difference indicated worse prognosis.

In this study, the need to perform decompression of bone flap indicated poor prognosis. As the decision to perform decompression of bone flap, depends on the patient's intracranial pressure, brain tissue edema, cerebral hernia, GCS, and other comprehensive factors. So it is logic to expect that the patients who underwent decompression of bone flap would have worse prognosis.

There were some limitations of this study. First, this was only a single-center retrospective study. Second, the operation time and method might also have impacts on the prognosis. Further, in-depth multicenter prospective studies with larger sample sizes are still needed in the future.

## 5 Conclusion

Nomogram model is the most effective factor to predict the prognosis of AEDH. The GCS, preoperative GWR, and postoperative GWR are closely related to the poor prognosis.

## Author contributions

In this study, all authors have made crucial contributions and agreed to publish the article. In particular, Dr. Yang played the leading role in the investigation. Meanwhile, Dr. He was mainly responsible for writing articles, Dr. Du and Dr. Jin were mainly responsible for data collection, statistical analysis, and other sections.

**Conceptualization:** Mingfei Yang.

**Data curation:** Yunxing Luo, Chaonan Du, Xiaoqing Jin.

**Formal analysis:** Yunxing Luo, Chaonan Du.

**Investigation:** Xiaoqing Jin.

**Methodology:** Xiwu He, Mingfei Yang.

**Project administration:** Mingfei Yang.

**Resources:** Yunxing Luo.

**Software:** Yunxing Luo.

**Writing – original draft:** Yunxing Luo, Xiwu He.

**Writing – review & editing:** Mingfei Yang.
